# CUTANEOUS REMODELING AND PHOTOREJUVENATION USING RADIOFREQUENCY DEVICES

**DOI:** 10.4103/0019-5154.55625

**Published:** 2009

**Authors:** Mohamed Lotfy Elsaie

**Affiliations:** *From the MBAFellow of Dermatology and Cutaneous Surgery, University Miami School of Medicine, USA.*

**Keywords:** *Radiofrequency*, *rejuvenation*, *thermage*

## Abstract

Radio frequency (RF) is electromagnetic radiation in the frequency range of 3-300GHz. The primary effects of RF energy on living tissue are considered to be thermal. The goal of the new devices based on these frequency ranges is to heat specific layers of the skin. The directed use of RF can induce dermal heating and cause collagen degeneration. Wound healing mechanisms promote the remodeling of collagen and wound contraction, which ultimately clinically enhances the appearance of mild to moderate skin laxity. Preliminary studies have reported efficacy in the treatment of laxity that involves the periorbital area and jowls. Because RF energy is not dependent on specific chromophore interaction, epidermal melanin is not at risk of destruction and treatment of all skin types is possible. As such, radiofrequency-based systems have been used successfully for nonablative skin rejuvenation, atrophic scar revision and treatment of unwanted hair, vascular lesions and inflammatory acne. The use of RF is becoming more popular, although a misunderstanding exists regarding the mechanisms and limitations of its actions. This concise review serves as an introduction and guide to many aspects of RF in the non ablative rejuvenation of skin.

## Introduction

Because of the high demand for antiaging treatments over the past decade, a proliferation of laser and light-based systems were developed. Nonablative technology replaced traditional ablative systems such as carbon dioxide and erbium:yttrium-aluminum-garnet lasers (long considered the criterion standards for skin resurfacing) because of the ability of these nonablative systems to induce dermal neocollagenesis without epidermal disruption (thereby limiting adverse effects and virtually eliminating postoperative recovery).[1–9] Because only modest success could be achieved with most of these systems, radiofrequency (RF) devices were introduced to address these shortcomings and to provide the added benefit of tissue tightening.[10–18]

## Radiofrequency in Medicine

Radiofrequency energy has become increasingly popular for medical applications that involve tissue heating in the fields of general surgery, cardiology, neurology, orthopedics and dermatology. It has been used for more than a century for a variety of medical applications, including tissue electrodesiccation and electrocoagulation,[19] joint capsular tightening,[20,21] corneal curvature alteration, incompetent saphenous venous closure,[22] aberrant cardiac electroconductive ablation[23,24] and prostate and liver neoplasm eradication.[25] The ability of RF energy to deliver heat to dermal structures results in nonsurgical lifting and tightening of tissue without the disruption of epidermal integrity.[26]

## Radiofrequency – The Basics

Radiofrequency current is formed when charged particles flow through a closed circuit. As the energy meets resistance in the tissue, heat is produced. The amount of heat will vary depending on the amount of current, the resistance levels in the targeted tissue and the characteristics of the electrodes. Human tissues, including the skin, are rich in electrolytes and an array of compounds that allow current conductance with varying degrees of impedance and resulting heat formation. The amount of RF energy applied can be configured to target specific tissues. In addition, the water content of skin varies between different areas of the body with time of the day, environmental humidity, internal hydration and the use of topical moisturizing agents. Thus, the flow of RF through the skin depends on multiple factors that may not be uniform from one treatment to the next. This reaction is dictated by the following formula: energy (*J*) = *I*^2^ × *R* × *T* (where *I* = current, *R* = tissue impedance and *T* = time of application). High-impedance tissues, such as subcutaneous fat, generate greater heat and account for the deeper thermal effects of RF devices.[27]

### Mechanisms of action

An underlying network of collagen and elastin fibers provides scaffolding for the skin and determines its degree of firmness and elasticity. Over time, this intricate fiber network loosens and unravels, altering the appearance and function of the skin. It is estimated that adult skin loses approximately 1% of its dermal collagen content on an annual basis due to increased collagen degradation and decreased collagen synthesis.[28] When the collagen fibers are heated, some of the cross-links are broken, causing the triple helix structure to unwind. Beyond a certain level, depending on a combination of both the maximal temperature and the exposure time, collagen fibers undergo denaturation. When the cross-links are maintained, at least partially, collagen shrinkage and thickening is achieved.[29]

Based on this principle, treatments are designed to cause the shrinkage of dermal collagen using heat generated by a radiofrequency current. In addition, the treatment promotes the formation of new collagen via the natural wound healing response of the skin and a direct effect on the dermal cellular matrix. The extent of collagen shrinkage, fibroblast activation, fibroplasia and overall collagenesis in the different skin layers is based on a complex multivariate mechanism, which depends on the temperature distribution and timing. This enables shrinkage at a certain depth, followed by collagenesis at a different, preferably more superficial, layer. Mechanical stress, (e.g, vacuum) has been reported to stimulate fibroblasts, leading to collagenesis. Notably, both heat exposure and application of vacuum to the skin are also known to increase blood perfusion in the affected area, supporting the fibroblast activity and the overall rejuvenation process.[30]

## Electrode Configuration

Two major electrode configurations are available in RF devices currently on the market – monopolar or bipolar. The two configurations differ in their derived energy field, but the resultant energy-tissue interaction is similar. In both cases, under controlled conditions, it is the tissue that becomes hot, not the electrodes. In a monopolar configuration, one electrode is active and the other (a considerably larger one) is placed far from the first one and serves as a grounding pad. The main advantage of monopolar delivery is the concentration of a high-power density on the surface of the electrode and the relatively deep penetration of the emitted power, making this configuration more suitable for electrosurgery. However, relatively high pain levels and safety concerns may be associated with utilization of this configuration for dermatologic applications. In a bipolar configuration, the current flows between two identical electrodes that are set at small fixed distance apart. No grounding pad is necessary. The fact that the distribution of the current in the tissue is more controlled in this setting is a major advantage over the monopolar configuration. However, in bipolar systems where the electrodes are placed flush on the skin, this configuration has the distinct disadvantage in that the depth of penetration is limited to approximately half the distance between the electrodes. This means that less energy of sufficient density reaches the deeper structures, rendering a more superficial effect regardless of the emitted energy level.[31]

Both monopolar and bipolar RF devices have been used for cutaneous applications. Monopolar systems deliver current through a single contact point with an accompanying grounding pad that serves as a low resistance path for current flow to complete the electrical circuit. Monopolar electrodes concentrate most of their energy near the point of contact and energy rapidly diminishes as the current flows toward the grounding electrode. Bipolar devices only pass electrical current between two positioned electrodes applied to the skin. No grounding pad is necessary with these systems because no current flows throughout the rest of the body. Monopolar RF devices such as the ablative Visage (ArthroCare Corp, Sunnyvale, Calif) and the nonablative ThermaCool TC (Thermage Inc, Hayward, Calif) and bipolar devices such as the Aurora and Polaris (Syneron Medical Ltd, Yokneam, Israel), have shown clinical utility within esthetic medicine for the treatment of excessive facial laxity and rhytide reduction, leg telangiectasias, acne and unwanted hair. In particular, these systems have proven most effective for the reduction of brow ptosis, prominent melolabial folds and cheek laxity.[2–5]

## Monopolar Radiofrequency

The nonablative RF devices deliver RF energy to the skin with concomitant contact cooling and are approved for the noninvasive treatment of facial rhytides by the US Food and Drug Administration. This system uses a high-frequency generator that produces a 330-W, 6-MHz monopolar current signal. A disposable membrane tip encompassing a treatment area of either 1.0 or 1.5cm^2^ is used with a disposable adhesive return pad that serves as the grounding point. The depth of heating is dependent upon the size and geometry of the treatment tip being used. A conductive coupling fluid is used during the treatment to enhance the thermal and electrical contact between the treatment tip and the skin.[32] This patented capacitive membrane tip allows for delivery of deep volumes of sustained, uniform and intense heat to tissue depths of 3-6mm. The treatment tip creates an electrical field within the tissue by alternating its charge from positive to negative 6 million times per second with electrons and ions simultaneously attracted and repelled from the surface. According to Ohm's law, it is the resistance of the tissue to the movement of these ions that generates heat.[33]

Transmission electron microscopy studies has shown immediate heating results in collagen denaturation with a resultant fibril contraction and tissue thickening.[32] An inflammatory wound healing response ensues with long- term neocollagenesis effecting rhytide reduction and further tissue contraction. In addition, selective heating and tightening of fibrous septae within the subcutaneous layer likely accounts for immediate contour changes in the skin after treatment.[33] Skin surface cooling is maintained before, during and after RF delivery through the use of a cryogen gas spray device. A balance of deep tissue heating and superficial cooling is therefore produced with creation of a reverse thermal gradient; the most intense heat is delivered deep within the dermis and subcutaneous layer while the superficial layers remain relatively unaffected by thermal delivery.

Several recent reports have demonstrated the safety and efficacy of RF delivery for rhytide reduction and tightening of lax facial and neck skin. Fitzpatrick *et al.*,[11] in the largest conducted study thus far, demonstrated an 83.2% improvement in periorbital rhytides and brow elevation of the patients enrolled. They also showed that patients who receive multiple RF treatment sessions had more impressive clinical results in term of rhytide reduction and improved skin tone in comparison to those receiving a single treatment.

An study conducted by Kushikata *et al*. in Asia[17] showed a gradual improvement in the lower cheek jowl and nasolabial folds after a single treatment with RF. Most of the 85 patients treated reported high satisfaction rates for the jowls, marionette lines and nasolabial folds at 3 months, but the scores slightly dipped 6 months after the treatment. The authors surmised that re-treatments would therefore best be performed within 5 to 6 months after the original treatment session for enhanced clinical results. It was also noted that the energy level selection with the device is best determined by constantly evaluating the level of pain tolerance for individual patients during the procedure. It should be noted that different skin compositions have different resistance and therefore will have a direct effect on the penetration and thermal deposition within tissues.

A promising use of RF in rejuvenation of the eyelids was also investigated.[34] Although the study was done on a smaller sample of patients, the authors reported a continuous improvement after treatment. Moderate to severe acne vulgaris was also targeted by RF studies.[35] Twenty-two patients received 1 or 2 RF treatment sessions with an observable dual benefit: both atrophic scarring and a reduction in active cystic lesions were noted. The authors hypothesized that RF delivery not only stimulated dermal remodeling eventuating in scar reduction but also directly inhibited sebaceous gland activity to improve acne.

### Guidelines for delivering RF treatments

Recommended treatment algorithms with the RF devices device have significantly changed since its introduction to the medical market place nearly 4 years ago. Initially, patients were treated with a single pass of the RF device at high-energy settings, often resulting in mixed clinical results and significant treatment discomfort. Newer treatment guidelines that use a multiple pass technique with reduced energy settings have been proposed, effecting superior clinical and histologic results as well as significant reduction in patient discomfort.[15,32] Although most practitioners initially delivered 100-150 pulses to the entire face and neck, current guidelines advocate the use of more than 400 pulses for the same areas. Patient feedback regarding tolerability is vital during treatment to avoid excessive thermal delivery to the skin. Although topical anesthetic preparations and oral anxiolytics help intraoperatively, caution should be used with complete and total anesthesia because of its tendency to reduce subjective patient feedback, which could potentially increase the risk of epidermal injury and subsequent thermal burns.[32]

Most patients experience mild erythema and edema, but these adverse effects usually subside within a few hours after treatment. There have been isolated reports of vesiculation and superficial burns after RF treatment, which have been attributed to operator error or use of excessive energy settings. Although improvement in skin laxity is not as pronounced as that observed with surgical lifting procedures, the advantages of RF procedures include a virtually nonexistent postoperative recovery period and extraordinarily low risk of serious adverse effects. Patients should be counseled preoperatively regarding the potentially modest results of the treatment despite the significant skin tightening often observed immediately after the procedure (due to immediate collagen contraction and tissue edema).

### Combined RF and optical energy

A unique combination of RF and optical energies, termed *electro*-*optical synergy*, has emerged in an attempt to address the limitations of traditional light-based systems.[16] The use of optical-based systems alone for skin rejuvenation and rhytide reduction has presented several challenges because tissue scatter and melanin absorption significantly decrease light penetration within the skin. Higher treatment energies are therefore required to adequately target dermal structures and this, in turn, increases the risk of integumental injury and potential for adverse sequelae.

This dual energy system has been used in photorejuvenation, hair removal, in treatment of leg veins, acne and sebaceous hyperplasia. A synergistic effect is obtained when the light energy component is used to heat the target tissue, which lowers impendance (tissues inherent resistance) of that tissue. This lowered impendance is used by the RF component to selectively heat the desired target. This synergy allows lower levels of both types of energy to be used, thus minimizing adverse effects such as blistering, burns and inflammatory nodules.[36]

The Aurora SR system is one of those dual energy systems and it uses intense pulsed light as its optical energy source with emissions between 400 and 980, 580 and 980, and 680 and 980 nm. The optical energy is emitted to preheat dermal structures, which then creates a temperature difference between the targeted structures and the surrounding tissues. It is used for skin rejuvenation, including rhytide reduction and improvement in skin texture and tone. Patients typically undergo 3 to 4 treatment sessions at 3- to 4-week intervals for rejuvenation. This system can also be used to treat facial acne or unwanted hair.[37,38] It is approved by the Food and Drug Administration for hair reduction and for the treatment of vascular and pigmented lesions. This system is also reported to remove lightly pigmented and white hairs because it does not rely solely on melanin absorption for target destruction.

Another dual-mode system using electro-optical synergy technology is *the Polaris WR system* – a combined 900-nm diode laser with RF energy device. Optical energies are delivered through a bipolar electrode tip with fluences ranging from 10 to 50 J/cm^2^ and RF energies from 10 to 100 J/cm^3^. These energies are simultaneously delivered to the tissue and while the RF energy penetrates more deeply and stimulates collagen production, the diode laser addresses superficial rhytides, pigmentation and vascularity. The two systems therefore work synergistically to treat deep wrinkles as well as the more superficial signs of photoaging.[14]

A recent study published by Doshi and Alster was the first to evaluate the dual-mode Polaris WR RF/diode laser system for wrinkle reduction and skin laxity.[14] Multiple laser passes were performed at each session, which were well tolerated. Evaluations 6 months post-treatment demonstrated modest improvements in wrinkles in most of the patients treated. Periorbital rhytides displayed greater improvements than that of the perioral rhytides in terms of mean clinical scores by end-study. There were no significant adverse effects and 80% of patients reported only mild treatment-associated discomfort.

## Conclusions

Nonablative skin rejuvenation with RF-based systems produces skin tightening through controlled dermal collagen contraction and neocollagenesis without integumental injury. This nonsurgical approach to rhytide reduction thereby avoids many of the inherent risks associated with surgical rhytidectomy. Experience with nonablative lasers and light sources has proven that tissue enhancement is possible with controlled dermal wounding without epidermal disruption. Radiofrequency devices are able to achieve greater depths of thermal injury with tissue penetration to the level of the dermis and subcutaneous layer without producing thermal burns. Tissue tightening and reduction of prominent nasolabial folds or jowling are produced as a result of this.

These systems have become very popular because of their minimal morbidity and low risk for postoperative complications. There have been rapid advances in RF technology over the past few years and the nonsurgical face or neck-lift using this energy source offers great promise to our aging population. Further studies are warranted to help elucidate ideal treatment settings particular to each system, identify the most appropriate candidates for treatment as well as to discover novel applications for RF energy within esthetic medicine.
